# Understanding the Barriers and Facilitators of Digital Health Technology (DHT) Implementation in Neurological Rehabilitation: An Integrative Systematic Review

**DOI:** 10.1177/11786329241229917

**Published:** 2024-04-29

**Authors:** Kathryn Jarvis, Clare Thetford, Edward Turck, Kelly Ogley, Rachel C Stockley

**Affiliations:** 1 Stroke Research Team, School of Nursing and Midwifery, University of Central Lancashire, Preston, UK; 2 School of Sport and Health Sciences, University of Central Lancashire, Preston, UK

**Keywords:** Digital health technology, implementation frameworks, rehabilitation, neurological conditions

## Abstract

**Background:**

Digital Health technologies (DHT) have potential to deliver intensive, novel and engaging rehabilitation for people with neurological conditions, yet health services lack a strong track record in embedding DHT into practice. The aim of this review was to synthesise factors that have been shown to influence implementation of DHT into neurological rehabilitation.

**Method:**

An integrative review was undertaken. An extensive search of MEDLINE, CINAHL, AMED, EMBASE was undertaken. The title and abstract of all retrieved sources were screened against pre-defined criteria. Retained sources underwent full text review. The quality of all included sources was assessed. A meta-ethnographic synthesis explored commonalities and contradictions of the included studies.

**Results:**

Fourteen studies (1 quantitative, 8 qualitative and 5 mixed methods) were included. Eleven implementation theories/models/frameworks were used across the 14 studies. Five themes were identified: (i) individual factors; (ii) user experience of the technology; (iii) the content of the intervention; (iv) access to the technology and (v) supporting use.

**Conclusions:**

Key factors which appear to influence the implementation of DHT into clinical settings are highlighted. Implementation theories, models and frameworks are under-utilised in DHT rehabilitation research. This needs to be addressed if DHT are to realise their potential in neurological rehabilitation.

**Registration:**

The protocol was registered and is available from PROSPERO (CRD42021268984).

## Background

Approximately 1 in 6 people experience a neurological condition,^1^ leading globally to an estimated 276 million years affected by disability (Disability Adjusted Life Years; DALYs)^2^ High intensity training elicits optimal functional and motor recovery following neurological injury,^3-6^ but this is often difficult to deliver in clinical practice.^7,8^ Digital health technologies (DHT), comprising a broad range of products including Applications (Apps), programmes and software,^9^ could have the potential to deliver intensive, novel and engaging rehabilitation for people with neurological conditions and are the focus of significant research endeavour. Common DHT used in rehabilitation include virtual reality gaming, electrical stimulation, robotics and telerehabilitation which may be used alone or combined with other products, including medical devices, such as brain computer interfaces.

Despite a clear potential to provide intensive interventions, health services lack a strong track record in embedding DHT into practice,^10^ with many DHT failing to be successfully used to deliver therapy for patients.^11^ Whilst the specific reasons for this widespread failure are not clear, it is likely that overt use of implementation processes and strategies will support and increase the adoption of DHT.^12^ Implementation theories, models and frameworks offer a means to systematically explore the translation of DHT-based interventions into practice, explore spread and provide a structure to explore the factors that influence both successful and failed adoption,^13^ However, it is unclear which implementation models, are used to support the implementation of DHT into rehabilitative practice and what factors are likely to have the greatest influence upon adoption despite this knowledge being vital if the promise of DHT to transform the outcomes for people with neurological conditions is to be realised. Therefore, the aim of this review was to synthesise factors that have been shown to influence implementation of DHT into neurological rehabilitation.

## Methods

An integrative review^14^ was undertaken. The protocol was registered and is available from PROSPERO (CRD42021268984).

### Search strategy

Following an extensive scoping search, search terms were identified based on 4 concepts: physical rehabilitation, neurology, implementation, and technology. The full search can be found in Appendix 1.

The following databases were last searched on 17th Jan 2023. MEDLINE (Jan 2006-date), CINAHL (Jan 2006-date), AMED (EBSCO) (Jan 2006-date), EMBASE (OVID) (Jan 2006-date). All database searches were restricted to English language publications. The searches were restricted to search the last 15 years, encompassing the first release of transformative digital technology such as the iPhone, and the advent of gaming consoles being used in rehabilitation.

### Selection of studies

All retrieved sources were transferred to reference manager software and duplicates removed. The remaining sources were transferred to Rayyan^15^ and screened according to a pre-defined selection criteria. Sources were included if they reported a primary study of a DHT implemented for neurological rehabilitation in a home, clinic or hospital setting with participants aged at least 18 years of age; used a defined approach to implementation (an implementation model, framework or outcome); explored at least one of the following: usability, feasibility, acceptability, barriers or facilitators, using qualitative or quantitative data collection methods; and were published in English language. Studies were excluded if the DHT was a diagnostic tool, or part of a surgical or invasive procedure; there were participants under 18 years of age and the data could not be separated for those of 18 years and older; only a protocol or abstract was available; the paper described only the development or theoretical context of DHT.

Two researchers (ET and KO) independently reviewed the title and abstract for a sample of 98 sources (2% of all those retrieved). Agreement was good with less than 10% conflicting decisions. These were subsequently agreed through discussion. The remaining sources were screened by only one researcher. Where there was uncertainty, the paper was discussed with the research team, and agreement reached. If agreement was not possible the paper was retained for full text review.

Three researchers (KJ, CT, RCS) independently reviewed the full text of the remaining papers. Where a study was not retained the reason for exclusion was documented based on a pre-defined exclusion code list. Any uncertainty about the suitability of a study was resolved through discussion with the wider research team.

### Quality assessment and data extraction

The quality of each study was assessed using the Quality Assessment with Diverse Studies (QuADS).^16^ This tool has been shown to be reliable and demonstrate content validity.^16^ It provides a score from 0 to 39, with a higher score indicating higher quality research. The quality of all papers was independently assessed by 2 of 3 researchers (RCS, CT, KJ). Discrepancies in score were discussed and a final negotiated score agreed.

Data extraction, into an Excel database, was completed by 1 of 3 researchers and checked by the research team. Extracted data comprised: the setting in which the DHT was used, sample characteristics, the type of DHT, intervention details, the frequency and duration of the intervention, and the implementation model, theory of framework underpinning the study.

### Data synthesis

Scoping searches indicated that the included sources would be diverse both in methodology and the DHT being studied. A meta-ethnographic synthesis^17^ was selected to enable an exploration the relationship between studies. This was achieved by following the established stages of this approach, ‘translating’ the studies onto each other, recognising areas of commonality (reciprocal synthesis) and differences or contradictions (refutational synthesis) which resulted in 7 phases^18^ (Table 1). The identified focus of the synthesis (Phase 1) was twofold. Firstly, it enabled exploration of the implementation models/frameworks used to underpin the implementation of digital technology, and to see how these theoretical frameworks have shaped the implementation processes. Secondly, it advanced our understanding of the experiences of those using the DHT and the barriers and facilitators to DHT use in clinical practice.

**Table 1.** Process through the 7 stages of the meta-ethnographic synthesis^17,18^.

Phase	What?	How?
1	Identified focus of synthesis	Informed by research aim: (i) to explore the implementation models/frameworks used to underpin the implementation of digital technology; (ii) to synthesise factors that have been shown to influence implementation of DHT into neurological rehabilitation
2	Selection of studies	Described in methods: Selection of studies
3	Identification of concepts and themes	Each included paper was analysed by two researchers, key concepts and possible themes noted. Consensus reached on factors influencing implementation of DHT through discussion.
4	Consideration of how studies related to each other	Three researchers considered the impact of the types of evidence/research designs/research aims and agreed through discussion how this should shape findings.
5	Translation of the studies	Three researchers reviewed the codes and preliminary themes to identify areas of commonality and differences or contradictions across the studies/different types of evidence. Changes to themes were agreed through discussion.
6	Synthesis of these translations	Two researchers undertook a final review of all identified factors that have been shown to influence implementation of DHT into neurological rehabilitation and the matured themes to ensure these reflected the evidence included in the review. This final synthesis was confirmed by the three researchers involved in the data synthesis.
7	Final synthesis produced	

Following selection of studies (Phase 2), 2 of the 3 researchers (RCS, CT, KJ) independently made decisions about what they considered of relevance, noting concepts and themes within the papers (Phase 3). Consideration of how the studies were related (Phase 4), and translating the studies (Phase 5), were agreed through discussions between all 3 researchers. Synthesis of these translations (Phase 6) was undertaken by 2 researchers (KJ and RCS) using a Padlet (https://en-gb.padlet.com/) to display an early analysis. This analysis was confirmed by all 3 researchers and a final synthesis produced (Phase 7).

## Results

**Figure 1.**
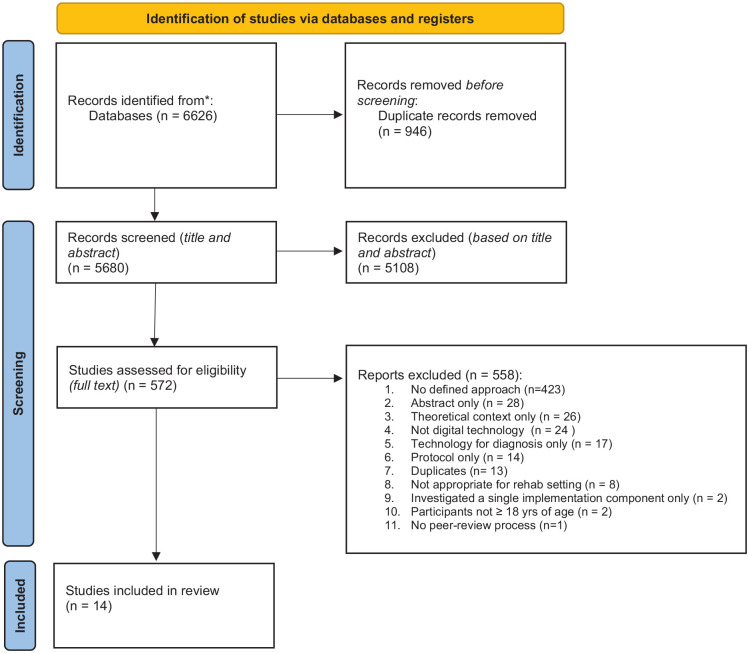
PRISMA flow diagram.

Fourteen papers were included for data extraction and analysis (Table 2). One was a quantitative study, 8 qualitative and 5 utilised a mixed methods approach. The results from the initial searches are accounted for in the PRISMA flow diagram (Figure 1).

**Table 2.** Summary of included studies.

Author	Setting	Study design	Implementation model, theory, framework	Sample	DHT intervention	How was the intervention delivered	Frequency and duration	Time spent using intervention	Quality tool score (QuADS) and main limitations
Andreassen et al^25^	Clinic and home	Mixed methods	Bowen’s feasibility framework	8 Outpatients: 6 male; 2 female median age 58 (26-68)	RemindMe – to support everyday activities using an interactive calendar and mobile phone reminders.	Patients given access to RemindMe and user profile created (30 min). Then patient and family received training and provision of written manual. Structured use of RemindMe – individual conversations with patients once a week (15 min) for 2 mo – with occupational therapists at clinic; or research assistant. Weekly conversations – support with reminders. Follow-up sessions at 2 and 4 mo (by researcher). No cost to participate.	Individual conversations between occupational therapist and patients once a week, for 2 mo. Reminders were set up for use (numbers/types varied according to individual need).	Not applicable	29 Limited by: sampling strategy, lack of recruitment data, and evidence of stakeholder involvement
				Cognitive impairment due to stroke (4/8); traumatic brain injury (2/8); sepsis (1/8); and multiple sclerosis (1/8).					
				All were used to using smartphones.					
				7 occupational therapiss; All female.					
Brouns et al^22^	Two rehabilitation centres (stroke rehab.)	Qualitative: 6 × focus groups with service-users and informal caregivers; 2 × focus group with healthcare professionals.		32 Patients, 15 informal caregivers, and 13 healthcare professionals patient inclusion criteria: (1) older than 18 y, (2) diagnosed with stroke and (3) completed rehabilitation which started after June 2011). Male: Patients 19/32; carers 4/15; staff 3/13 Mean (sd) age: patients 56.9 (15.1); carers 60.6 (9.9)	e-Rehabilitation (not clear if participants had actually experienced e-rehabilitation)	Not applicable	Not specified	Not specified	29 Limited by: insufficient theoretical approach, and evidence of stakeholder involvement
Brouns et al^21^	Two rehabilitation centres – stroke rehabilitation	Cross-sectional study-quantitative online survey	Grol’s implementation model	125 Stroke patients, 43 informal caregivers and 105 healthcare professionals	e-Rehabilitation (not all participants had used/participated in e-Rehabilitation)	Not applicable	Not specified	Not specified	28 Limited by: insufficient theoretical approach and description of the setting, and lack of justification for analytical method
				Male: Patients 72/125; carers 16/43; staff 25/102					
				Mean (sd) age: patients 58.2 (11.4); carers 58.4 (12) staff 41.9 (10.6); Mean time since stroke was 30.6 mo (SD 29.2).					
Brouns et al^32^	Specialised rehabilitation unit, inpatient and outpatient rehabilitation	Mixed methods: process evaluation	Process evaluation based on Medical Research Council Framework	Patients registered for fast@home n = 165	Fast@home is a web-based eRehabilitation intervention consisting of already existing (commercially available) eRehabilitation applications: physical exercise programme, cognitive exercise programme, physical activity tracker and psycho-education	Web-based, e-rehabilitation, accessed through personal computers and devices	Patients instructed to use Fast@home 5 times a week for 30 min, for 16 wk.	Not specified	28 Limited by: insufficient description of the setting, limited justification for data collection/analytical methods, and evidence of stakeholder involvement
				Staff invited to participate in survey n = 80					
				Patients registered: *Male* = 103/165, 62.8%; of those participating in process evaluation *Male* = 43/73, 58.9%.					
				Those participating in process evaluation mean age of 62.9 (SD 13.2); Staff: Male = 3/11%, 27.2%, 3 OTs and 8 PTs.					
Buckingham et al^29^	Not stated	Service evaluation: data collected through on-line or telephone discussions	The Knowledge to Action (KTA) framework	21 Practitioners, 7 patients, 2 carers took part in individual discussions. In addition, 4 group discussions were held with 23 practitioners	Telerehabilitation	Not specified	Not specified	Not specified	29 Limited by: insufficient description of the setting, and limited justification for data collection/analytical methods
				Practitioners Individual discussions: physician n = 4, nurse n = 1, occupational therapist n = 4, physiotherapist n = 10, podiatrist n = 1 social worker n = 1					
				Group discussion: dietitian n = 23)					
				Patient diagnoses: cystic fibrosis, multiple sclerosis, musculoskeletal condition. Parkinson’s disease, stroke, post-COVID					
				Male/female: practitioners 9/35; patients 4/3; carers 0/2					
Celian et al^27^	Rehabilitation hospital	Qualitative: phenomenological approach − 5 therapists wrote vignettes explaining their rehabilitation technology use decisions during treatment sessions with 9 patients.	Consolidated Framework for Implementation Research (CFIR)	3 occupational therapists, 2 physiotherapists	Neurorehabilitation technology	Not applicable	Varied – range of interventions	Varied interventions	26 Limited by: sampling strategy, limited justification for data collection/analytical methods and lack of evidence of stakeholder involvement
				Male/Female Not reported					
Chen et al^19^	Home	Qualitative study design, embedded in a clinical trial; In-depth semi-structured interviews	Unified Theory of Acceptance and use of tech (UTAUT)	13 Stroke survivors (2 female/9 male)	Telerehabilitation system-four main components: games, exercises, education, telecommunication. The system delivers treatment sessions in the form of daily guided rehabilitation games, exercises, and stroke education.	Members of the research team delivered the telerehabilitation system to the subject’s home, set it up, confirmed functionality, and reviewed use of the system with the subject. Patients were assigned a guided rehabilitation programme using the system for 70 min at a fixed time every day, 6 d per wk, over 6 to 8 wk.	70 min at a fixed time every day, 6 d per week, over 6 to 8 wk.	Not specified	13 Limited by: insufficient theoretical approach inadequate sampling strategy, limited details of, or justification for data collection/analytical methods, lack of evidence of stakeholder involvement and critique of study limitations
				Stroke survivors (9 were accompanied by a carer)					
				Average age of participants 70.52. Seven R-sided stroke, 6 L-sided stroke					
Christensen et al^31^	Specialised neurorehabilitation centre	Qualitative: interviews (study phase), group discussion (act phase)	Quality improvement model with an implementation model Plan-Do-Study-Act (PDSA) and Consolidated Framework for Implementation Research (CFIR)	N = 9	Armeo®Spring robotic technology	Presume face-to-face-not stated	Not stated	Not stated	25 Limited by: sampling strategy, limited justification for data collection/analytical methods, lack of evidence of stakeholder involvement
				Therapists for interviews. Other staff involved in Act and Plan, but barriers/facilitators based on interviews; Interview stage: therapists who had been trained and had used Armeo®Spring					
				Interviews-4 physiotherapists and 5 occupational therapists (all women, age: 35-47 y)					
				Participants were employed in average for 11.1 y (range 6-17 y). Trained in the use of Armeo®Spring in average 4.7 y ago (range 2.5-6 years)					
				Group discussion-4 ward managers, the head of therapists at the hospital, 3 researchers and 5 therapists, distributed across 6 wards, participated-no demographics given.					
Hochstenbach-Waelen and Seelen^23^	Stroke rehabilitation centre and children’s rehabilitation	Qualitative: literature review and semi-structured interviews.	Grol’s implementation model	7 occupational therapists and physiotherapists	Implementation of technology	Not applicable	Not applicable	Not applicable	18 Limited by: sampling strategy, limited details of, or justification for data collection/analytical methods, lack of evidence of stakeholder involvement and critique of study limitations
				Male/female Not reported					
Nguyen et al^20^	Hopsital exergaming room	Qualitative: interviews – staff that have referred patients to the exergame room	Unified Theory of Acceptance and use of tech (UTAUT)	10 Clinicians (92% female) from the Stroke Programme	Virtual reality exergaming	Referring therapists defined the specific goals for the patients that they referred. Once they were referred another therapist chose the games they used OR the referring therapist could choose the games. The patient was supported by an assistant to complete the games once a week.	Following the evaluation, an assistant worked with client on a weekly basis to complete the exergame programme established by the expert clinician.	Not specified	27 Limited by: insufficient theoretical approach inadequate sampling strategy, limited recruitment data provided, and lack of evidence of stakeholder involvement
				6 Occupational therapists (6 female); 4 physical therapists (1 male, 3 female)					
				All had referred patients to the exergame room in the last 12 mo					
				Mean age of 34.5 y old; between <1 and 25 y working in neurology					
Stockley and Christian^28^	Inpatient rehabilitation	Qualitative: focus group	Theoretical Domains Framework (TDF)/Capability, Opportunity, Motivation-Behaviour (COM-B) behaviour change model	4 Therapists. 2 × occupational therapists and physiotherapists working in acute stroke rehab 3 female, 1 male. Experience ranged from 4.5 to 15 y	Neuroanimation virtual reality game	Face to face with patients undergoing rehabilitation after stroke in an inpatient acute rehabilitation setting. Delivered technology as part of a trial (Neuroanimation training = VR) intensively for an hour a day.	Daily training 5 d a week for 3 wk, 1 h time on task plus usual rehab.	1 h time on task (but longer to complete this time with rest breaks etc)	34 Limited by: insufficient justification for data analytical method
Teriö et al^24^	Home	Mixed methods: single case study, semi-structured interviews, quantitative process data.	Integrated Promoting Action on Research Implementation in Health Services framework (i-PARIHS)	12 Participants; 4 occupational therapists; 3 researchers; 3 IT specialists; 2 rehabilitation. managers	F@ace: Mobile phone supported, family-centred rehab intervention	Mobile phone – SMS and calls	8 wk of intervention. 3 set activity targets delivered by SMS every morning and evening. Morning SMS reminder to perform exercise through the day. Evening SMS client to respond to 3 messages to score the activities. If no response or score 0, occupational therapist would call client. Clients also recieved phone calls from occupational therapist ×2/wk.	Not specified	27 Limited by: insufficient recruitment data, limited justification for data collection/analytical methods, and evidence of stakeholder involvement
				Stroke patients/occupational therapists/researchers/service managers/IT specialists					
				Gender not stated					
Yang et al^26^	Home	Mixed methods	Reach, Effectiveness, Adoption, Implementation and Maintenance (RE-AIM) framework	17 stroke patients screened; 13 eligible; 11 consented; 9 included 4 female; 5 male	GRASP: Virtual Graded Repetitive Arm Supplementary Programme for individuals with stroke during COVID pandemic.	Group classes via videoconferencing using Zoom, with individual instructor, supported by 2 to 3 volunteers. Patients (and carers) at home, using a range of devices to access. 2 virtual GRASP programmes delivered	10 weekly 1 h group sessions on Zoom, Supplemented with 1 h homework exercises	10× 1 h	36 Limited by: lack of evidence of stakeholder involvement
				Average age 65.9 (SD 14.4); range 39 to 83. 3 Asian, 4 white, 2 European, 7.9 to 348 mo post-stroke (mean 65.86 mo); 2 patients had aphasia;					
Zarshenas et al^30^	Supported community residence-a charitable organisation that provides support and housing for adults with acquired brain injury	Mixed methods: multiple baseline, single case study	Fit between Individuals, Task, and Technology, Environment (FITTE)	N = 1 participant with acquired brain injury. Female, 47 y, 10 y post-stroke	Cognitive Orthosis for coOKin (COOK) COOK. Comprises: (1) a sensor-based monitoring security system to monitor safety incidents; (2) a cognitive assistance application to increase independence providing step by step instructions in the process of meal preparation; and (3) a configuration system that makes it possible to tailor COOK’s features to the individuals’ needs while providing accessibility to a COOK log (e.g., type of errors in following safety rules)	COOK app on tablet. Sensors on cooker. Staff supported use.	Intervention phase (acquisition, application, adaption) Minimum: 2 wk, 2 sessions/wk for an hour. Maximum: could be tailored to the client’s needs. Followed by 6 wk follow-up phase	Not stated	27 Limited by: insufficient theoretical approach, research design as a means to answer research question, and lack of evidence of stakeholder involvement
				Care team, a behavioural therapist, a personal support worker and a coordinator, who work closely with participants with ABI					


### Implementation theory and frameworks

Eleven different implementation theories/models/frameworks were used across the 14 studies: Unified Theory of Acceptance and Use of Technology (UTAUT)^19,20^; Grol’s implementation model^21-23^; Integrated Promoting Action on Research Implementation in Health Services framework (i-PARIHS)^24^; Bowen’s feasibility framework^25^; Reach, Effectiveness, Adoption, Implementation and Maintenance (RE-AIM) framework^26^; Consolidated Framework for Implementation Research (CFIR)^27^; Theoretical Domains Framework (TDF)/Capability, Opportunity, Motivation-Behaviour (COM-B) behaviour change model^28^; Knowledge Transfer Approach (KTA)^29^; Fit between Individuals, Task, Technology and Environment (FITTE)^30^; Plan-Do-Study-Act (PDSA)^31^; Medical Research Council (MRC) Process Evaluation.^32^

These theories/models/frameworks were utilised in study design,^25,26,29,31,32^ data collection,^20,21,24-26,28,30,32^ data analysis.^19,22-24,26-28,30,32^

### Types of DHT

Ten papers studied a single DHT. These comprised telerehabilitation (3 studies),^19,26,29^ Apps (2 studies),^25,30^ and virtual reality (2 studies).^20,33^ Robotics,^31^ a web-based programme^32^ and a telephone supported rehabilitation^24^ were each the focus of 1 study. Four papers^21-23,27^ took a broad approach, acknowledging a range of DHT.

The quality of the papers was assessed using the QuADS.^16^ Papers were assessed and scored out of a maximum of 39 points. Overall, the majority of papers included in the review were of moderate quality (with scores in mid to high 20s; 10 papers). Two papers were assessed to be of high quality (scores in mid 30s); and the remaining 2 papers were of poor quality (scores under 20).

### Themes

Following initial data synthesis (phases 1-5) 6 themes were established. Once Phase 6 of the analysis was complete, these were reduced to 5 themes and 9 sub-themes. One theme related to the individual or person, 2 themes to the DHT and 2 themes to the environment, described in Table 3.

**Table 3.** A summary of the themes, theme descriptions and sub-themes.

	Themes	Description of theme	Sub-themes
The person	Individual factors	Includes: capability (knowledge, skills, abilities), experience, beliefs, motivation of individuals interacting with the DHT	Patient factors Staff factors
The DHT	User experience of the technology	Factors that contribute to the experience of using the DHT-this theme is focussed on the hardware and software of the DHT	
	The content of the intervention	How the DHT is being used to provide therapy	Adaptability of the intervention Practical considerations Enabling interactions
The environment	Access to the technology	Practical factors that influence the opportunity to use the DHT (incl. safety)	
	Supporting use	Support required to enable access and use of the technology	Patient/therapist interactions Supporting use of the technology Clinical team Training

### The person

One theme (*Individual factors*) and 2 sub-themes (Patient Factors; and Staff Factors) focussed on the person using the DHT.

#### Patient factors

An individual’s perceived benefit of the DHT on recovery^20,21,24,30^ and an individual’s experience or technology habits^20,22,25,29^ were the most frequently reported patient factors. Motivation to change^24^ and a willingness to try a different approach^27^ were recognised as facilitators, whilst fatigue, ataxia, pain, wheelchair-use, cognitive status, cognitive deficits and limited movement were identified as potential barriers to patient engagement.^20,27,31^ Staff, patients, and informal carers recognised that DHT-based rehabilitation programmes are not appropriate for all patients.^22,29^

#### Staff factors

Staff were influenced by the benefit to the patient^28^ alongside a perception that the use of technology enabled evidence-based practice.^32^ However, the most frequently reported personal factors affecting the staff adoption of DHT was having sufficient skills and knowledge to ensure that they had the expertise and confidence^20,22,24,27,29^ to use the technology. Previous experience of technology and staff beliefs about whether the DHT could meet patients’ needs were also perceived to impact staff adoption.^27,32^

### The DHT

Two themes (User Experience of the Technology; and Content of Intervention) and 3 sub-themes (Adaptability of the Intervention; Practical Considerations; and Enabling Interactions) related to the DHT and its properties.

#### The user experience

An easy log-in process, set-up and use^22-24,27,32^ were identified as enablers, along with the need for clear instructions and quick familiarisation.^23^ Technical characteristics^26^ such as the stability, reliability and system performance of the technology^20,22,23,28,32^ were also clearly identified as important to enable access and adoption. Hochstenbach-Waelen and Seelen^23^ identified that it is beneficial to staff if the DHT is portable and if the system is ‘invisible’ so that it does not detract from the therapy.

Patients appreciated being able to use the technology in their own home^29^ and staff appreciated being able to observe patients in this environment, indicating that this helped their understanding.^29^ It was suggested that DHT should ideally facilitate independent use^23^ and where support is required, it should be easy to involve family/carers^29^

#### The content of the intervention

##### Practical considerations

Staff and patients identified that DHT design should be engaging.^19,27^ Staff identified DHT as a useful adjunct to face-to-face therapy,^29^ but recognised that it needs to integrate with current therapy provision.^32^

##### Adaptability of the intervention

There was a recognition that DHT needs to have the capacity to be tailored^22,23,27,30^ and be adaptable to patient’s physical and cognitive needs,^19,21,23^ and designed with consideration of cognitive and communication impairment.^23,24^ There should be capacity to increase the difficulty of the task,^23^ building task-related skills rather than compensatory strategies.^23^

The technology should have overt goals^23^ and should provide feedback to both patient and therapist and show progress over time,^19,23^ ideally producing objective data.^27^ The DHT needs to be modifiable to fit the context^20^ and applications or games need to be varied and adaptable to ensure they are challenging and motivating.^19,23^

##### Enabling interactions

The DHT appeared to have an important role enabling interactions. Brouns et al^21^ reported that eRehabilitation was an easy way to communicate and continue contact with staff after discharge. The opportunity for peer contact,^22^ and an alternative means for consultations^21,22^ were also seen as a positive influence on the adoption of DHT.

### The environment

Two themes (Access to Technology; and Supporting Use) and 4 sub-themes (Patient/Therapist Interactions; Supporting Use of the Technology; Clinical team; and Training) related to the environment in which the DHT was used.

#### Access to technology

A range of factors affecting access to the DHT were identified. Insufficient financial resources^21,22,25,26^ impacted internet connection,^22,25^ availability of health insurance to cover intervention costs^22^ and access to the technology.^24,27^ Unwieldy processes to protect expensive equipment were also cited as a barrier.^27^

Making the technology available across a range of settings,^25^ accessible on multiple devices^24^ and outside of standard therapy delivery^22^ were reported to support accessibility. Where the DHT was physically large, in addition to the physical space requirement,^19,22,27-29^ it was important that the room was always set up ready for the DHT to be used.^20^ There was no indication that DHT was perceived to save time. Time was required to enable therapists to learn how to use the technology,^32^ and to plan^20,29^ and deliver an intervention using the technology.^19,22,27,28,32^ Depending on the technology, the therapist may also be required to provide the supervision to ensure safety during use.^20^

Organisations had to meet their legal and organisational requirements before providing access to a technology. They had to be confident the DHT adhered to data protection requirements^22,23^ and that infection control measures could be put in place.^28^

#### Supporting use

The relationship between the patient and therapist was seen to influence the adoption of DHT, with patients using DHT appreciating regular conversations with staff.^25^ Studies identified the need to develop the patient/therapist relationship to access and support the use of the technology,^25,27^ with a recognition that some patients needed more support than others.^28^ The role of the therapist in monitoring the activity undertaken by patients using the DHT was also identified as an influencer as this accountability provided motivation to the patient.^19,22^

#### Supporting use of the technology

Human support to use the DHT was recognised as a facilitator for the patient and the therapist, with support from a helpdesk,^21,22,32^ local facilitators,^20^ technical advisers and digital champions,^19,22,28,29^ family and friends,^19^ carers, and trained volunteers^29^ all being reported.

#### Clinical team

Therapists gained support from therapists/clinical champions,^26,32^ with 2 papers identifying the benefit of reminders to staff to encourage them to refer for^25^ and to use^20^ the technology. Interactions within the clinical team were seen as influencers^25^ enabling the sharing of practice and finding ‘workarounds’ through problem-solving.^29^ These interactions were evident where teams had established lines of communication^24^ and a positive work culture.^31^ The introduction of DHT was also found to facilitate cooperation between occupational therapists and physiotherapists.^32^ Two papers recognised the role of healthcare management in supporting the clinical team to introduce the DHT.^31,32^

#### Training

Eight papers identified the importance of training to ensure staff have sufficient skills and knowledge to be confident using the DHT^20,22,24,27,28,30-32^ with a means to maintain competency when the DHT is not used.^31^ The training should be provided by experts^30^ and should include opportunity to practice using the technology.^31^ A manual or resource for refreshing knowledge was also seen to support use of DHT.^28,31^

## Discussion

This review sought to determine the factors that influence the implementation of DHT in neurological rehabilitation to inform therapist, researcher and developer stakeholders. Only papers that utilised a defined approach to implementation were included which meant that the majority of retrieved studies (558 papers, 90%) were excluded because they did not articulate a distinct implementation theory, model or framework. This highlights a worrying under use of implementation strategies to support the adoption of DHT and emphasises the need to consider implementation approaches both in research and practice. From the 14 included papers, facilitators and barriers that are likely to influence implementation into clinical practice were identified. These encompassed the individual, the DHT and the environment. All except 2 studies^21,22^ reported the experience of implementing technology, giving confidence that the themes and sub-themes capture key factors that have previously affected adoption. Future studies should investigate these factors, for example using mixed method studies and process evaluation to understand real-world impact, and capture additional facilitators and barriers that have not yet been identified. Whilst many of these key factors are likely to be shared across different types of DHT, we do recognise that some individual DHT may have distinct factors that influence their implementation. However, in the absence of sufficient evidence, and an adequate taxonomy which articulates the features of DHT beyond a technology type, it was not possible to be more nuanced in our approach.

The 14 studies utilised 11 different implementation theories, models, and frameworks, highlighting a diversity of approaches to technology implementation. Some studies used theories or frameworks (eg, UTAUT) that were specific to technology adoption but which did not consider the wider context in which the technology will be used nor the needs of the users, despite these factors being likely to influence the success of sustained adoption in clinical practice.^11^ Other studies did use implementation models and frameworks that considered the training and motivation of users, vital to promote the sustained engagement with technology necessary to benefit from rehabilitation (eg, CFIR and COM-B). However, it was beyond the scope of these frameworks to reflect the distinct, unique demands of technology-based interventions which limits their usefulness.

These omissions and the range of the theories, models, and frameworks used by studies in this review underscores a pressing need for a comprehensive model for DHT implementation in rehabilitation. The review themes indicate that this comprehensive model should reflect and capture: (i) factors that affect patient and staff engagement, (ii) user experience of the technology, (iii) specific characteristics and content of the technology, including the ability to tailor the technology to meet patients’ complex needs and to change behaviour to encourage repeated engagement with challenging activities over many weeks, (iv) patient and staff access to the technology, (v) support required to use the technology both at a service level, including the physical space and training, and more broadly within the organisational context. It should be noted that a few frameworks, not used by studies in the current review, do reflect some of these characteristics,^11,34^ but to our knowledge, there is no single framework or theory that captures the particular demands of rehabilitation technologies, despite an exponential growth in their use in the last decade. The reasons for this are not known, but it is likely to be, at least in part, due to the novel, varied nature, and relatively rapid proliferation of DHT in rehabilitation. The multifaceted interactions required between developers, researchers, clinicians and patients to support implementation and may also account for a delay in developing a unified framework.

The complex and precarious process of implementing a DHT has been summarised as ‘a long and fragile chain of events’^27^ (p. 9). The findings of our review, notably the influence of personal factors (staff and patient), features of the technology (the content and the user experience) and the environment (access to, and support to use the technology) upon implementation supports this and highlight a range of factors which should be considered when implementing DHT into clinical settings.

Further research is now required to gain a deeper understanding of the factors that influence implementation. There is a clear necessity to develop an implementation model that can be used to support the implementation of DHT for rehabilitation; this model should consider the themes identified in the current study to provide a comprehensive guide to DHT implementation.

Further work could also consider the roles and features of different forms of DHT which present distinct implementation challenges from both a user and organisational perspective. Whilst others have provided definitions for telehealth for people after stroke,^35^ there is currently no lexicon to adequately describe the requirements of many DHT despite forms of the same classification of DHT presenting diverse demands upon users (eg, non-immersive commercial gaming versus fully immersive, rehabilitation-focussed forms of virtual reality). A taxonomy which includes the requirements, demands and benefits for forms of DHT would be helpful to enable precision descriptions in future studies and support implementation planning. Whilst this work is still to be undertaken, researchers of current DHT studies can utilise the TIDieR checklist^36^ to enable better understanding of the features of the technology and how it was used to support and deliver DHT.

More widely, research and development of DHT for rehabilitation should consider implementation theory, models and frameworks to plan for clinical implementation from the outset. This will enable systematic identification and understanding of the factors that influence successful implementation of DHT in practice, increase the chance of successful adoption and enable patients to benefit from the use of DHT in their rehabilitation.

### Strengths and limitations

In this study we have used a recognised integrative review methodology^14^ to synthesise qualitative and quantitative findings from primary studies. The coding and synthesising of these data was inevitably subjective; however, a reflexive approach combined with 2 researchers independently data extracting, analysing and assessing quality, mitigated these threats to rigour.

There may have been novel facilitators and barriers described in papers that were excluded from this review due to the lack of a defined implementation approach. Whilst this was a limitation, we made the decision to exclude these papers as we could not be confident about their applicability or influence upon implementation because they were not articulated clearly or contextualised with a theory, model or framework. Future research in this area would benefit from a defined approach to implementation to describe and analyse the implementation strategies.

The findings of our review have emerged from heterogeneous studies with a range of participants, included clinical staff, patients and carers, and variety in the DHTs. Whilst the review conclusions need to be viewed in this context, the recurrence of the themes across the studies provides confidence that these findings provide credible insights into the factors that influence implementation of DHT into neurological rehabilitation.

## Conclusion

The findings of this integrative review highlight a range of factors which should be considered when implementing DHT into clinical settings if DHT are to realise their potential to revolutionise neurological rehabilitation. It also highlights that implementation theories, models and frameworks are under-utilised in DHT rehabilitation research, signifying a lack of systematic approach to the introduction of technology, despite successful adoption into practice being vital to confer benefits to patients. From those studies that did utilise a recognised approach to implementation, 5 themes and 9 sub-themes describing the influence upon the implementation of DHT for neurological rehabilitation were identified: person/individual factors (patient factors, staff factors); user experience of the technology; the content of the intervention (adaptability of the intervention, practical considerations, enabling interactions); access to the technology; and supporting use (patient/therapist interactions, supporting use of the technology, clinical team, training). It is not possible to prioritise these factors; each should be considered during implementation planning, as any one might influence the success of adoption. Collectively, these factors are not considered in one model of implementation, suggesting that development of a comprehensive model for DHT adoption in rehabilitation should be a future focus of research. Thorough understanding of the key factors likely to influence DHT adoption into rehabilitation would also support overt consideration of implementation of DHT through the technology life cycle, ensuring that DHT are designed and developed, from the outset, to be implementable into clinical practice. We believe that this review makes an important initial contribution to our understanding of DHT implementation in rehabilitation by synthesising current knowledge and highlighting key facilitators and barriers.

## Supplemental Material

sj-docx-1-his-10.1177_11786329241229917 – Supplemental material for Understanding the Barriers and Facilitators of Digital Health Technology (DHT) Implementation in Neurological Rehabilitation: An Integrative Systematic Review Supplemental material, sj-docx-1-his-10.1177_11786329241229917 for Understanding the Barriers and Facilitators of Digital Health Technology (DHT) Implementation in Neurological Rehabilitation: An Integrative Systematic Review by Kathryn Jarvis, Clare Thetford, Edward Turck, Kelly Ogley and Rachel C Stockley in Health Services Insights

sj-docx-2-his-10.1177_11786329241229917 – Supplemental material for Understanding the Barriers and Facilitators of Digital Health Technology (DHT) Implementation in Neurological Rehabilitation: An Integrative Systematic Review Supplemental material, sj-docx-2-his-10.1177_11786329241229917 for Understanding the Barriers and Facilitators of Digital Health Technology (DHT) Implementation in Neurological Rehabilitation: An Integrative Systematic Review by Kathryn Jarvis, Clare Thetford, Edward Turck, Kelly Ogley and Rachel C Stockley in Health Services Insights
