# Supplementary material for: Understanding the Barriers and Facilitators of Digital Health Technology (DHT) Implementation in Neurological Rehabilitation: An Integrative Systematic Review
Source: Health Serv Insights. 2024 Apr 29;17:11786329241229917. doi: 10.1177/11786329241229917 (PMC11060031; doi:10.1177/11786329241229917)
Supplement: sj-docx-1-his-10.1177_11786329241229917 – Supplemental material for Understanding the Barriers and Facilitators of Digital Health Technology (DHT) Implementation in Neurological Rehabilitation: An Integrative Systematic Review [file sj-docx-1-his-10.1177_11786329241229917.docx]

Appendix 1

Search Terms

| **Group** | **Keywords** |
| --- | --- |
| **Physical Rehab** | physiotherapy  OR "physical rehabilitation" OR "physical therapy"  OR "physical therapy modalities"  OR "physiotherapy techniques"  OR "physiotherapy rehabilitation"  OR "physical therapist"  OR physiotherapist  OR "physical therapy modalities" OR "physical therapy specialty" OR "physical and rehabilitation medicine" OR rehabilitation OR rehabilitative OR neurorehabilitation OR "neurological physiotherapy" OR "neurological rehabilitation" OR "stroke rehabilitation"  OR mobilisation OR "activities of daily living" OR "functional activities"  OR "functional training"  OR "exercise therapy"  OR ambulation OR mobility OR "functional assessment"  OR "exercise therapy" OR "recreation therapy" OR "early ambulation" OR "exercise"  OR "recovery of function" OR "occupational therapy"  OR "allied health worker" OR "allied health professional"  OR "occupational therapist" OR "rehabilitation centre"  OR "occupational therapy" OR "allied health occupations"  OR "therapeutics"  OR "physical therapist assistants"  OR "allied health personnel" OR "geriatric rehabilitation" OR "recreational therapy" OR "vocational rehabilitation" OR "speech and language therapy" |
| **Technology** | telemedicine OR "virtual reality" OR "vr" OR "video games" OR "microcomputers" OR "biomedical technology"  OR "electronics" OR "mobile applications"  OR "technology" OR "telemedicine" OR "tele-medicine"  OR "biomedical technology assessment" OR "digital health" OR ehealth OR e-health OR "health technology" OR mhealth OR m-health OR "mobile health" OR "patient portals" OR "remote consultation" OR "remote sensing technology" OR "wearable sensor " OR "teleconsulting" OR "tele-consulting" OR telehealth OR telerehabilitation OR "text messaging" OR "wearable technology" OR "wireless technology" OR xbox OR "kinect" OR wii OR nintendo OR playstation OR "games consoles" OR iphone OR ipad OR "android tablet" OR smartphone OR "app" OR "virtual rehabilitation" |
| **Implementation** | barrier*  OR facilitat*  OR imped*  OR challenge  OR hinder*  OR obstacle*  OR enabl*  OR enabler OR perspect*  OR "delivery of care" OR implement*  OR adopt*  OR facilitate* OR feasibility OR qualitative OR "mixed methods" OR usability OR "considerations for use" OR "cohort study" OR "interview" OR accessibility OR accept* OR mixed-method OR "technology implementation" OR "technology acceptance" OR "technology accessibility" |
| **Neurology** | "neurological disease" OR "neurological degeneration" OR "neurological" OR stroke or strokes or "stroke" or "brain infarction" or "brain infarctions" or "cerebrovascular disorder" or "cerebrovascular disorders" or "cerebrovascular disorders" or "cerebrovascular accident" or "cerebrovascular accidents" or "cerebral infarction*"  or "brain infarction" or "intracranial embolism and thrombosis" or "cerebrovascular disease" or "cerebrovascular diseases" or "cerebral haemorrhage" or "cerebral hemorrhage" or "cerebral hemorrhage" or "brain ischemia" or "brain ischaemia" or "cerebral ischemia" or "cerebral ischaemia" or "brain ischemia" |
